# Publisher Correction: A pipeline for malignancy and therapy agnostic assessment of cancer drug response using cell mass measurements

**DOI:** 10.1038/s42003-022-04376-8

**Published:** 2023-01-06

**Authors:** Robert J. Kimmerling, Mark M. Stevens, Selim Olcum, Anthony Minnah, Madeleine Vacha, Rachel LaBella, Matthew Ferri, Steven C. Wasserman, Juanita Fujii, Zayna Shaheen, Srividya Sundaresan, Drew Ribadeneyra, David S. Jayabalan, Sarita Agte, Adolfo Aleman, Joseph A. Criscitiello, Ruben Niesvizky, Marlise R. Luskin, Samir Parekh, Cara A. Rosenbaum, Anobel Tamrazi, Clifford A. Reid

**Affiliations:** 1 Travera, Medford, MA USA; 2 Department of Clinical Research, Dignity Health, Sequoia Hospital, Redwood City, CA USA; 3 Weill Cornell Medicine, New York, NY USA; 4 Department of Medicine, Hematology and Medical Oncology, Icahn School of Medicine at Mount Sinai, New York, NY USA; 5 Tisch Cancer Institute, Icahn School of Medicine at Mount Sinai, New York, NY USA; 6 Graduate School of Biomedical Sciences, Icahn School of Medicine at Mount Sinai, New York, NY USA; 7 Department of Medical Oncology, Dana-Farber Cancer Institute, Boston, MA USA; 8 Precision Immunology Institute, Icahn School of Medicine at Mount Sinai, New York, NY USA; 9 Department of Oncological Sciences, Icahn School of Medicine at Mount Sinai, New York, NY USA; 10 Division of Vascular and Interventional Radiology, Palo Alto Medical Foundation, Redwood City, CA USA

**Keywords:** Tumour biomarkers, Assay systems, Predictive markers

Correction to: *Communications Biology* 10.1038/s42003-022-04270-3, published online 26 November 2022.

The original version of this Article contained a formatting error in Fig. 1C, in which the example images for the CNN classification are not displayed properly. The correct version of Fig. 1C is:
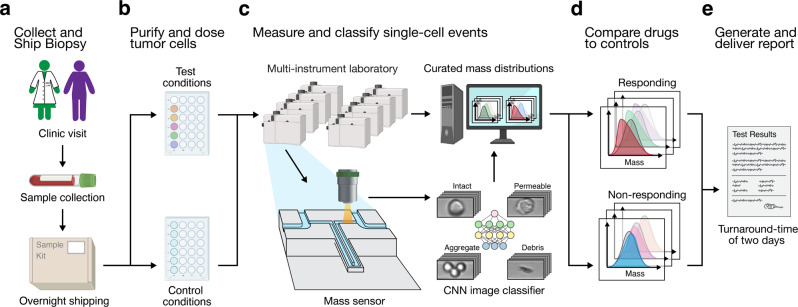


which replaces the previous incorrect version:
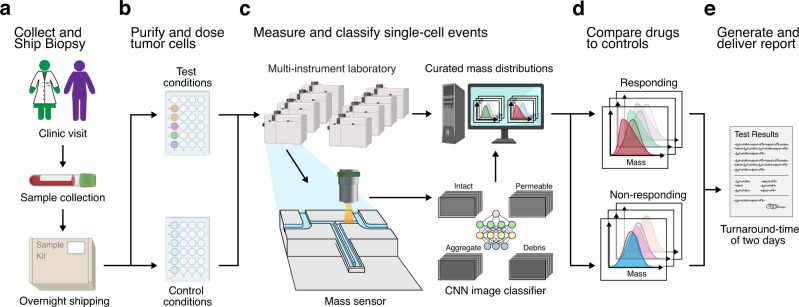


This has now been corrected in both the PDF and HTML versions of the Article.

