# Supplementary material for: A pipeline for malignancy and therapy agnostic assessment of cancer drug response using cell mass measurements
Source: Commun Biol. 2022 Nov 26;5:1295. doi: 10.1038/s42003-022-04270-3 (PMC9701192; doi:10.1038/s42003-022-04270-3)
Supplement: Supplementary file 2 — Supplementary Information [file 42003_2022_4270_MOESM2_ESM.pdf]

Supplementary Information

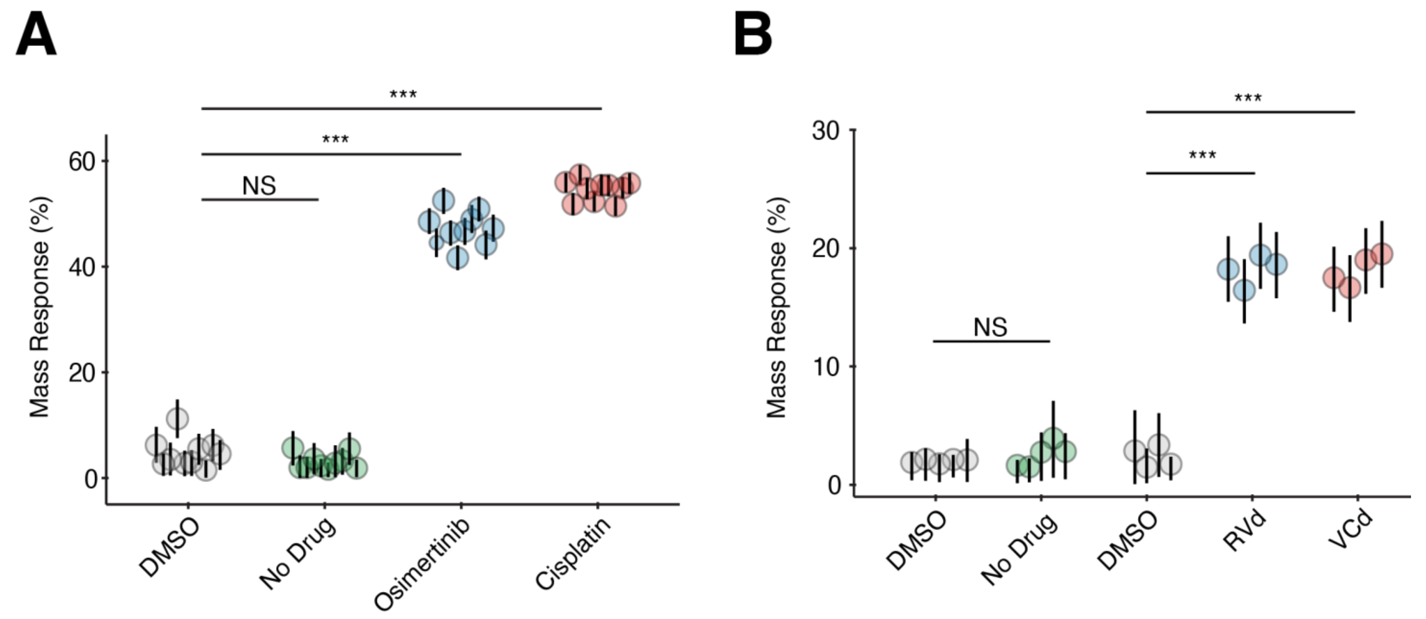

Supplementary Figure 1| Technical reproducibility of mass response measurements

(a) Mass response measurements collected on 10 different SMR instruments (individual points) for PC9 cells treated with vehicle control (DMSO), no drug, osimertinib (250nM), or cisplatin (100μM) for 20h. (b) Mass response measurements collected on 5 instruments (left) for MM1S cells treated with vehicle control (DMSO) or no drug, and 4 instruments (right) for MM1S cells treated with vehicle control or either a combination of 10μM lenalidomide, 50nM bortezomib, and 2uM dexamethasone (RVd) or 50nM bortezomib, 10μM mafosfamide, and 2uM dexamethasone (VCd) for 15h. Both experiments demonstrate the assay concordance expected across multiple instruments. Error bars on each point indicate the 95% confidence interval for Mass Response (%) measurements. *** indicates p<0.001, ns indicates p>0.05.

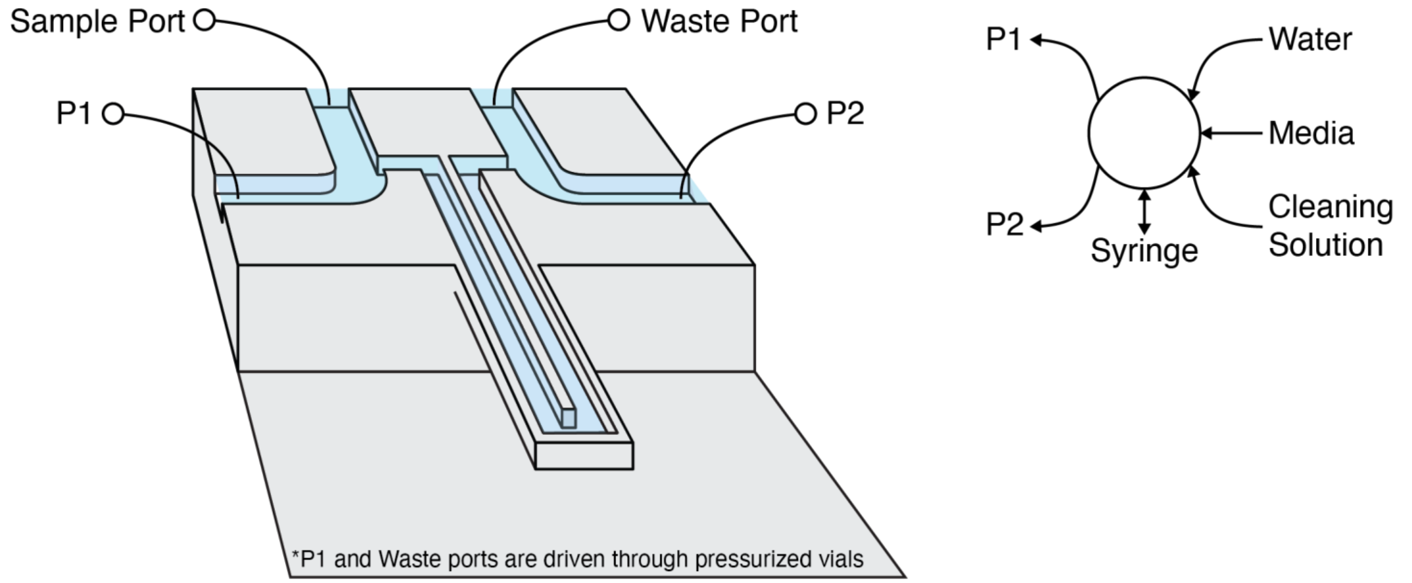

Supplementary Figure 2| SMR fluidic schematic

The SMR chip utilizes 4 fluidic ports for flow control. Two bypass channels, each connected to a pair of ports, flank the SMR mass sensor. Because the flow in each bypass channel can be controlled independently, this structure enables steering a population of cells through the mass sensor continuously with minimum sample loss. The port upstream of the first bypass channel (Sample Port) is used to introduce the sample to chip with pressure driven flow, while the ports downstream of the second bypass channel (P2) and downstream of the first bypass channel (P1) are used to introduce cleaning solutions between sample runs using syringe pump driven flow. The port upstream of the second bypass channel (Waste Port) is used as a waste outlet. During sample measurement, flow is stopped at ports P1 and P2 and the rate of flow across the SMR mass sensor is determined by the pressure drop between the Sample Port and the Waste Port. This ensures that the entire sample volume is flown through the mass sensor without any volumetric loss to P1.

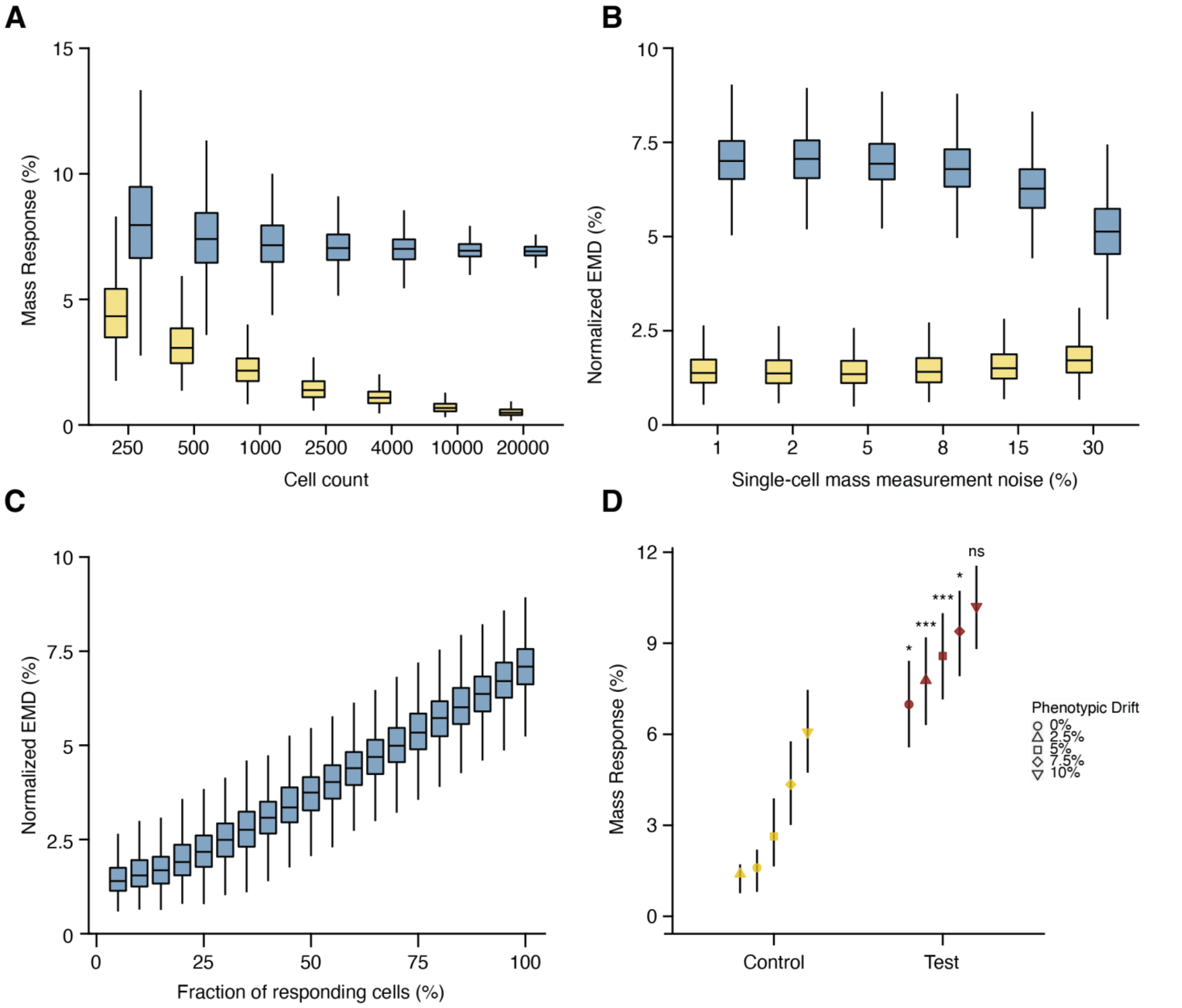

Supplementary Figure 3| Mass response simulation results

(a) The impact of the number of cells measured on mass response. Panel shows the calculated mass responses when a varying number of cells are randomly sampled 100 times from cell populations of a million cells with distributions shown in **Figure 2**. Blue boxes show the mass response when cells that are drawn from the blue *test* distribution and the grey *reference* distribution shown in **Figure 2** are compared. Yellow boxes show the mass response when cells that are drawn only from the *reference* distribution are compared. **(b) The impact of mass measurement noise on mass response.** Panel shows the calculated mass response when 2,500 cells are compared 100 times with varying magnitudes of single-cell mass measurement noise. The x-axis is normalized with the mean mass of the reference population. The compared distributions are identical to those in **(a)**. **(c) Mass response is proportional to the fraction of responding cells in a sample.** Test populations are created by randomly sampling from the reference and test distributions shown in **Figure 2** at varying ratios. All test populations are compared to reference populations sampled from only the reference distribution shown in **Figure 2**. **(d) The impact of phenotypic drift on mass response.** Different magnitudes of phenotypic drift are simulated in the form of single-cell mass loss as a function of time. The phenotypic drift is introduced to reference, test and control cells that are randomly sampled from the distributions shown in **Figure 2**.

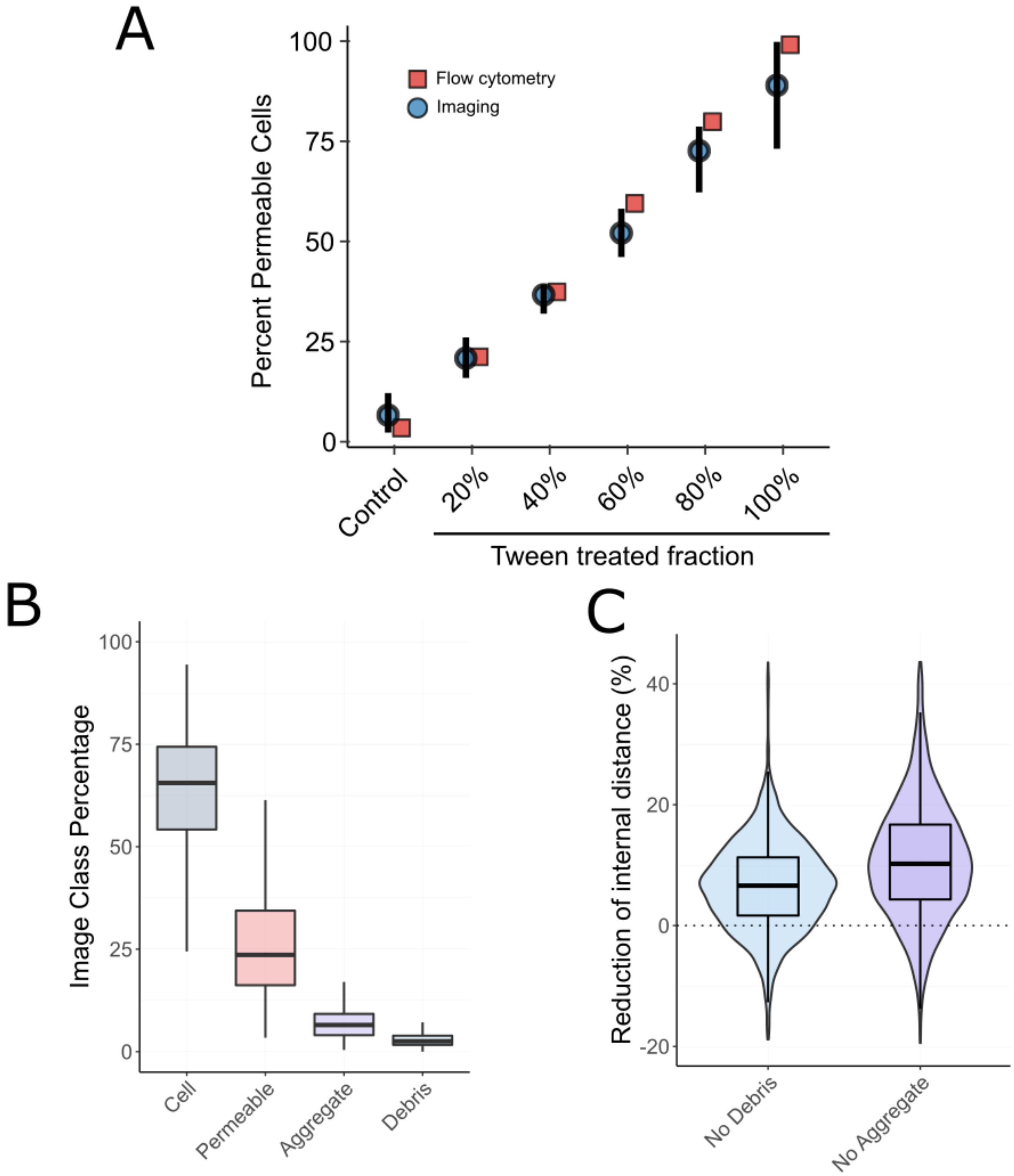

Supplementary Figure 4| Characterizing image classification performance

(a) Plot comparing the fraction of cells found to be DAPI+ (indicative of cell permeability) by flow cytometry versus the fraction of cell images collected on the SMR instrument classified as permeable for murine leukemia cells (L1210) either unperturbed (control) or mixed with various fractions of cells treated with 0.5% Tween 20 for 10 min to induce permeability. Images were collected for three different SMR instruments, bars overlaid on points indicate range of values measured across all instruments. **(b)** Boxplot showing the fraction of events classified as each particle type (Cell, Permeable, Aggregate, Debris) for all the experiments presented in **Figure 3c**. **(c)** Violin plots with overlaid boxplots showing the reduction of internal distance improvement offered by excluding either debris (left) or aggregates (right) alone from mass response analysis. While the results presented in panel's **(b)** and **(c)** demonstrate that the fraction of aggregate events was typically low for the samples presented here, it is important to note that drug response measurements collected from cell populations with high fractions of cellular aggregates processing may be difficult to interpret is using the image classification scheme presented here.

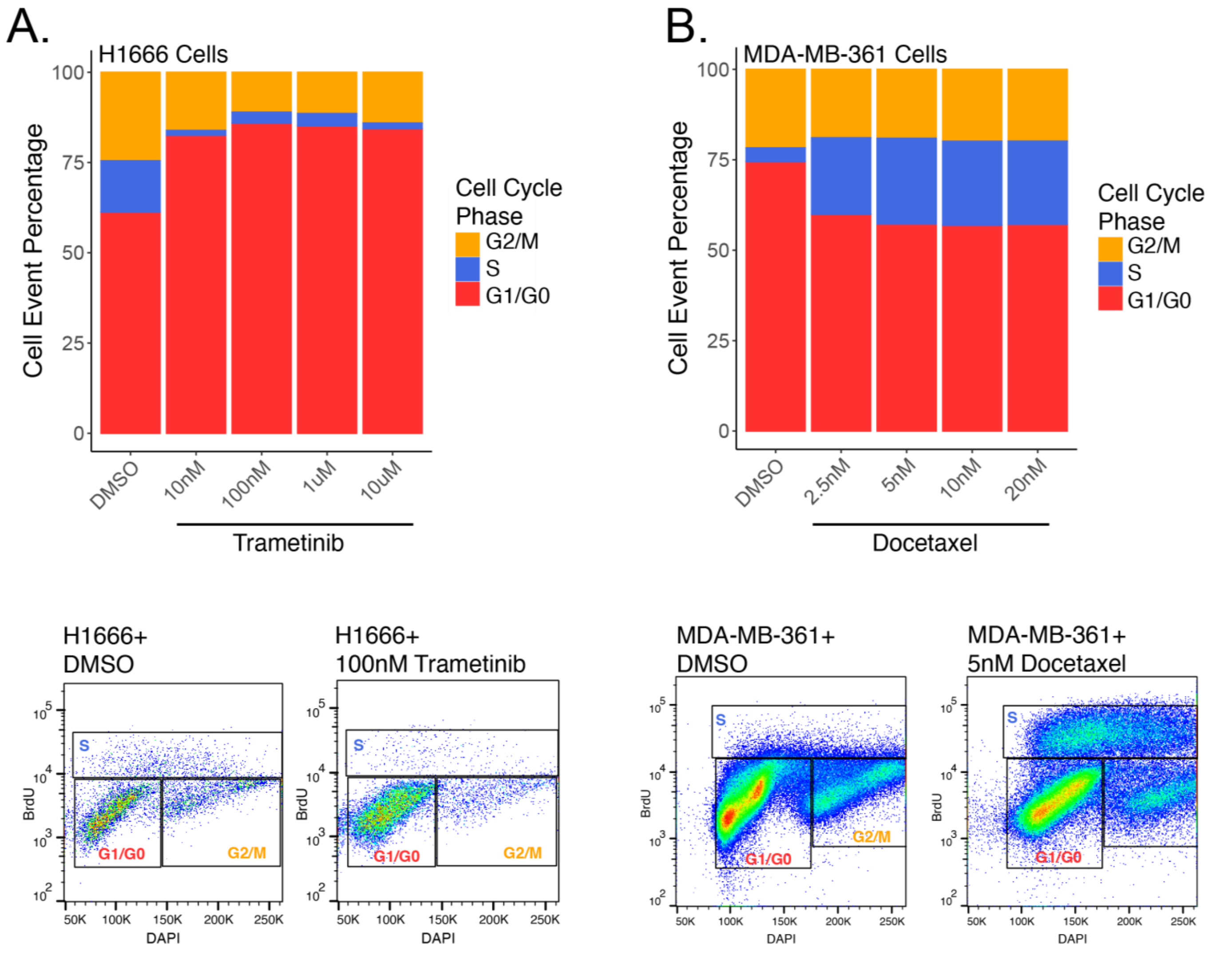

Supplementary Figure 5| Cell cycle arrest verification with flow cytometry

Cell cycle phase (G1/G0, S, G2/M) quantification determined with flow cytometric assessment of BrdU incorporation as a bar plot (top) as well as representative flow cytometry dot plots (bottom) showing DAPI assessment of DNA content on the X axis and BrdU quantification on the Y axis for (a) H1666 cells (human lung cancer) treated with varying concentrations of trametinib for 17h leading to early cell cycle arrest (as indicated by an increase in the fraction of G1/G0 cells) and (b) MDA-MB-361 cells (human breast cancer) treated with varying concentrations of docetaxel for 24h leading to late cell cycle arrest (as indicated by a decrease in the fraction of G1/G0 cells).

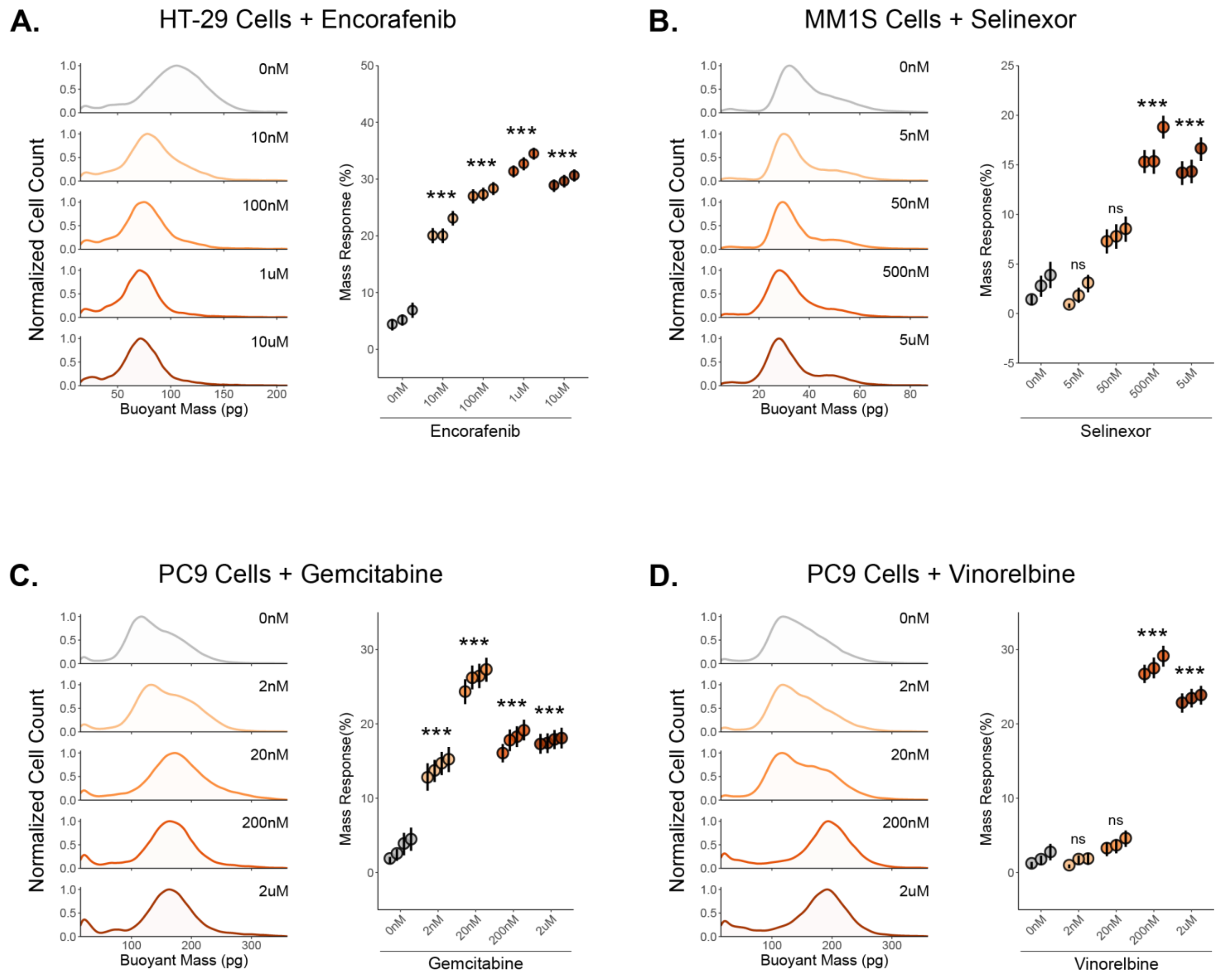

Supplementary Figure 6 | Additional examples of mass response measurements collected for drugs with arrestive MOAs

Mass distribution changes (left) and mass response measurements (right) for drugs with different arrestive mechanisms. (**a,b**) G1 arrest in HT-29 cells treated with encorafinib for 24h or MM1S cells treated with selinexor for 15h. (**c,d**) G2/M arrest in PC9 cells treated with gemcitabine or vinorelbine for 24h. *** indicates $p<0.001$, ns indicates $p>0.05$. Points in mass response plots indicate independent instruments, error bars indicate 95% confidence interval for mass response.

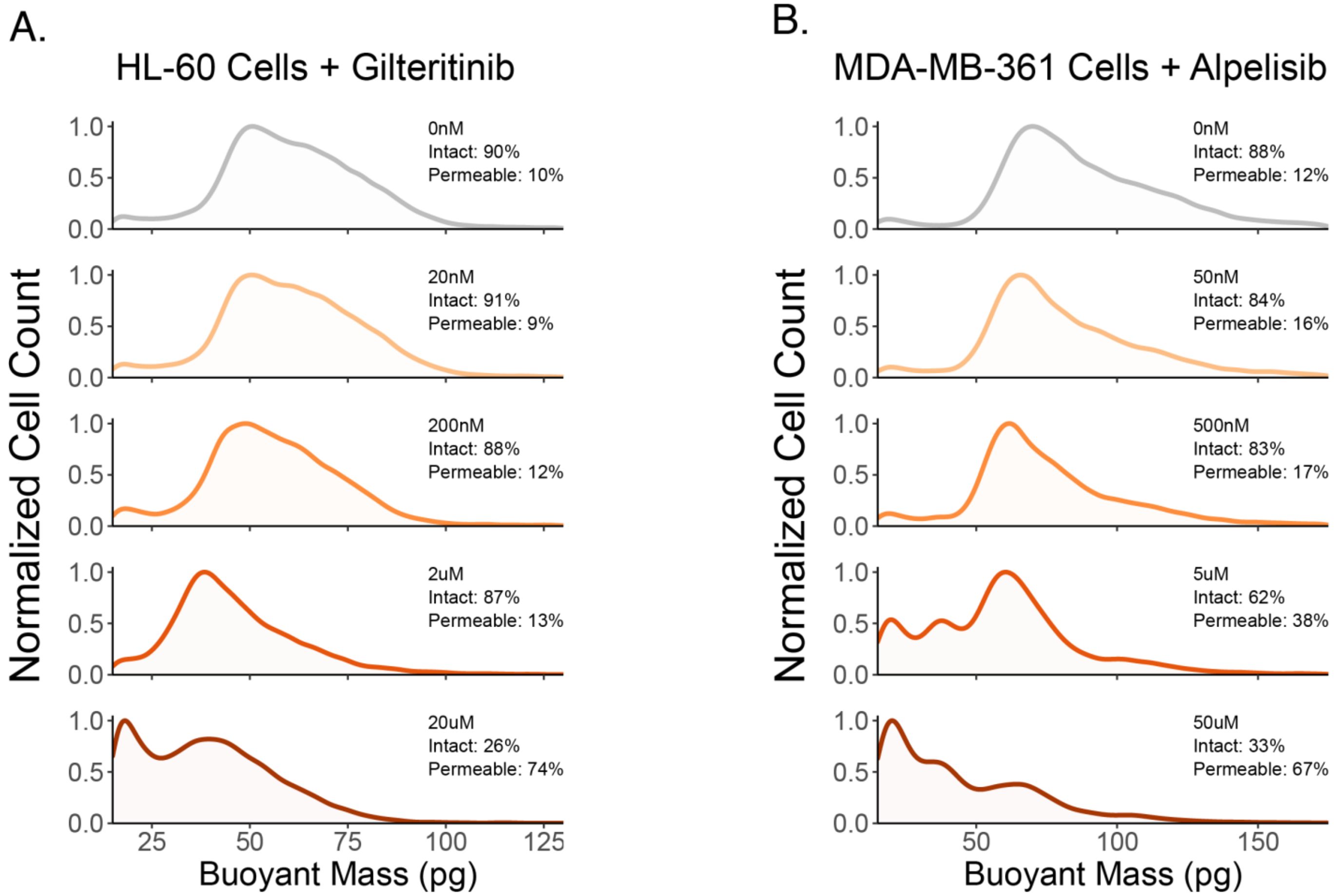

Supplementary Figure 7 | Additional examples of cell death corresponding to a significant loss in cell mass

Mass distribution changes for drugs that cause loss of cellular structural integrity. (**a**) HL-60 cells treated with gilteritinib for 15h. (**b**) MDA-MB-361 cells treated with alpelisib for 24h. Plot insets indicate the fraction of cell images that were classified as either intact or permeable for the corresponding mass distribution.

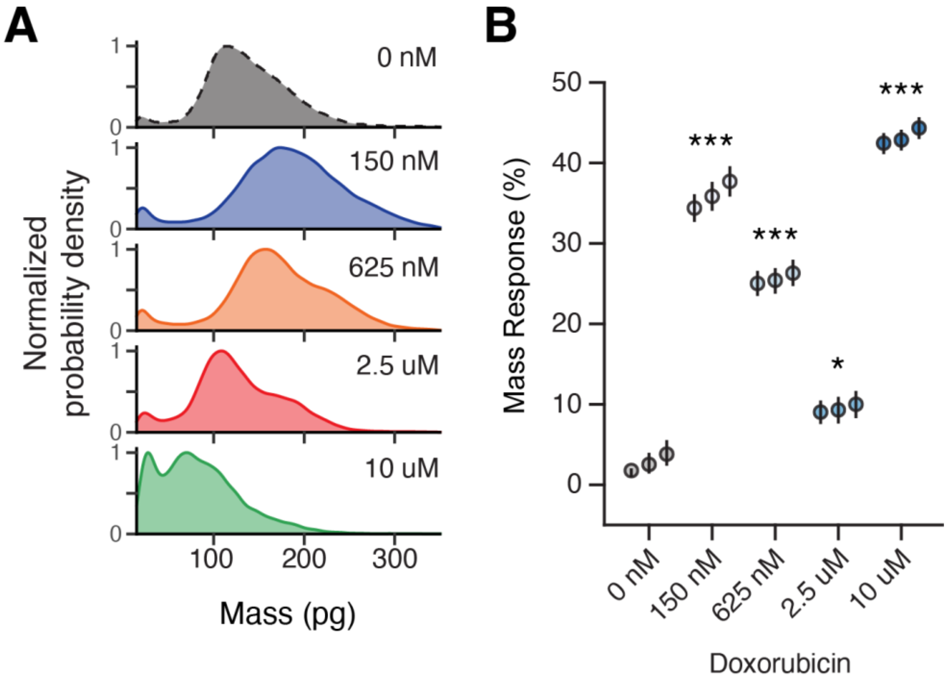

Supplementary Figure 8 | Mass response results for PC9 cells treated with doxorubicin for 24h

Mass distribution plots (left) and mass response plot (right) for PC9 cells treated with various doses of doxorubicin for 24h. *** indicates $p<0.001$, * indicates $p<0.05$. Points in mass response plot indicate independent instruments, error bars indicate 95% confidence interval for mass response.

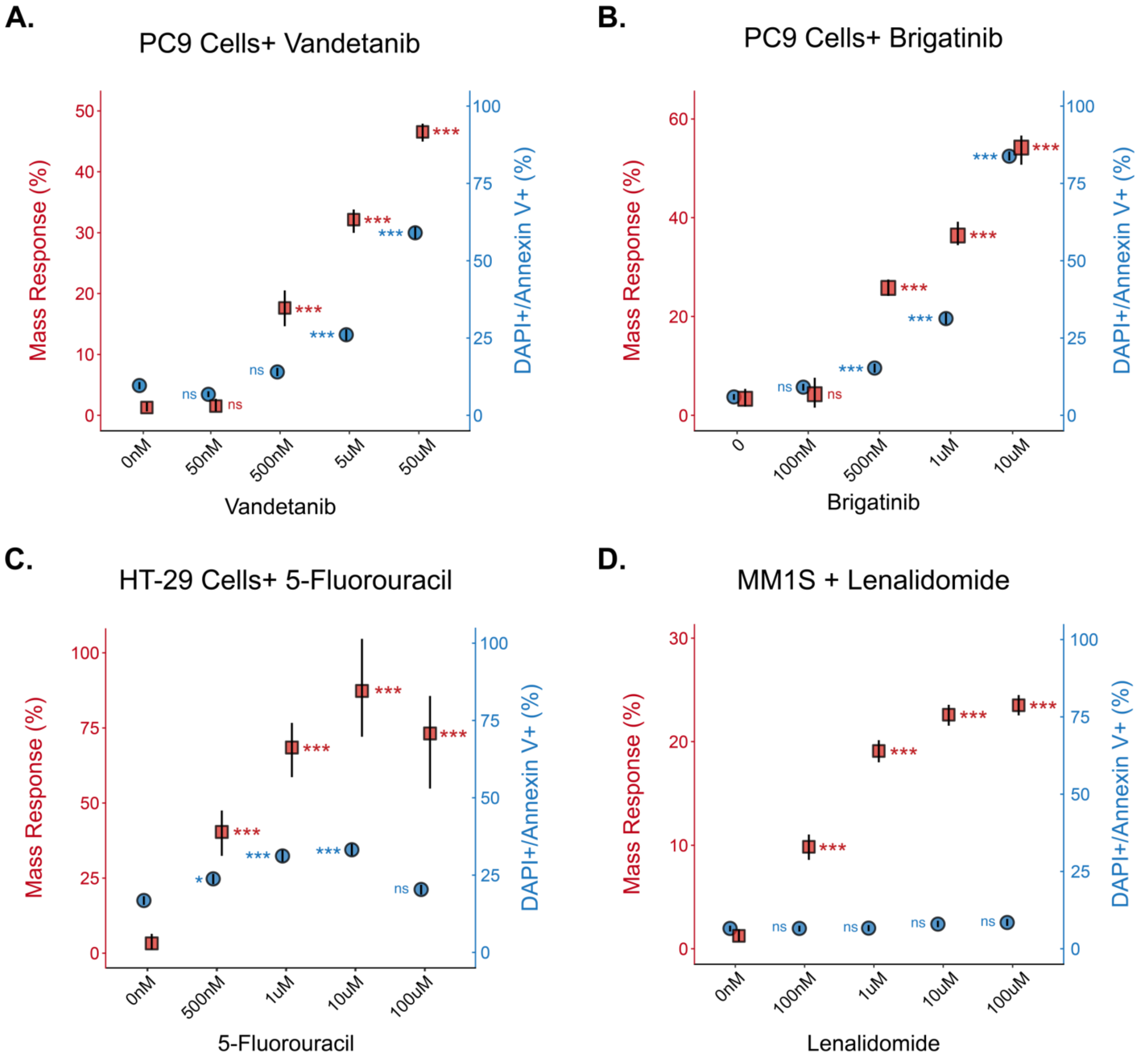

Supplementary Figure 9| Mass response versus cell viability for fast and slow acting drugs

Plots showing cell viability determined by flow cytometry (blue circles, fraction of cells displaying either DAPI or Annexin V positivity, overlaid bars indicate 95% confidence interval) and mass response (red boxes, Earth Movers Distance as a percentage of the reference cell population mass, overlaid bars indicate 95% confidence interval) for **(a)** PC9 cells (human lung cancer) treated with varying concentrations of vandetanib for 20h (n = 3 SMR instruments), **(b)** PC9 cells treated with varying concentrations of brigatinib for 20h (n = 3 SMR instruments), **(c)** HT-29 cells (human colon cancer) treated with varying concentrations of 5-fluoroacil (n=4 SMR instruments), and **(d)** MM1S cells (human multiple myeloma) treated with varying concentrations of lenalidomide (n=1 SMR instrument). Bars overlaid on square points indicate range of mass response measurements collected across all SMR instruments, where applicable. Fast acting drugs (**a** and **b**) show consistent trends between viability and mass response measurements whereas slow acting drugs (**c** and **d**) show significant mass responses that precede any significant measurable loss of viability. *** indicates p <0.001 and ns indicates p>0.05.

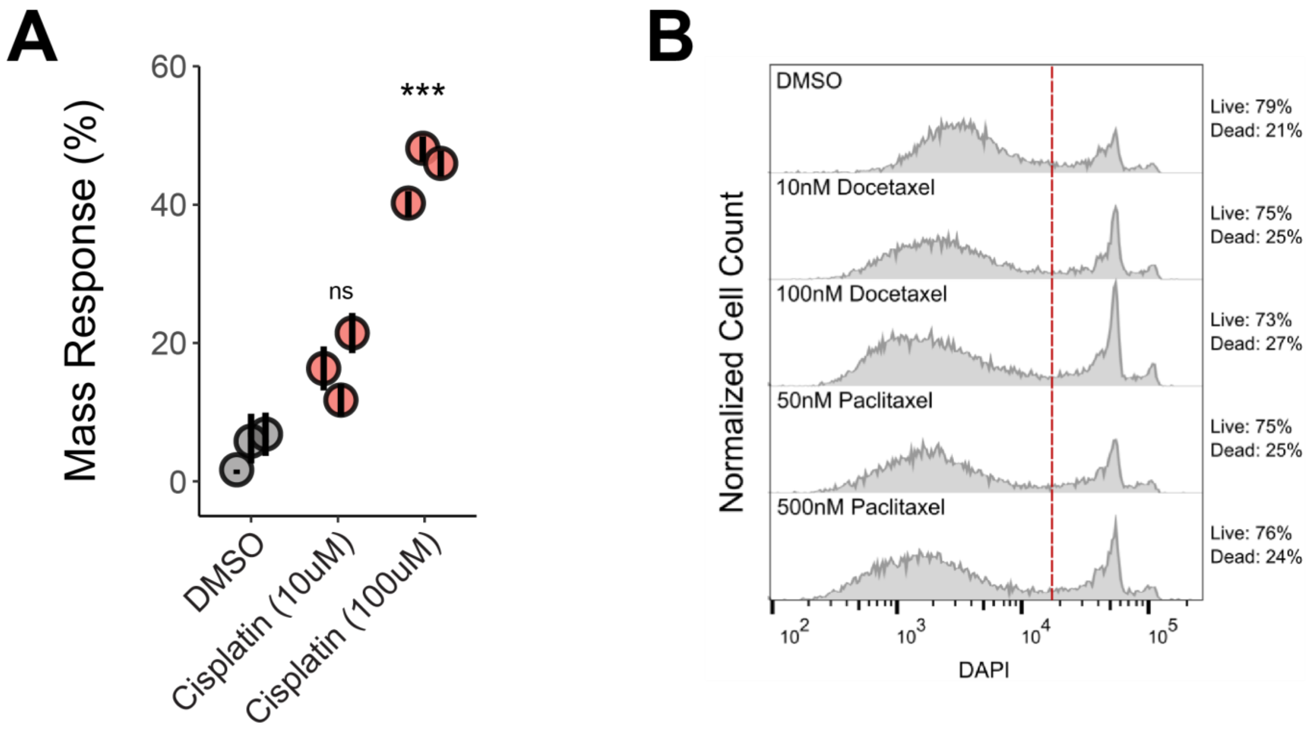

Supplementary Figure 10| Primary pleural effusion drug response

Additional drug response data for the NSCLC pleural effusion specimen presented in **Figure 7** showing **(a)** mass response to different concentrations of cisplatin and **(b)** flow cytometry-based viability measurements (as determined by DAPI, dotted red line indicates threshold for determined live versus dead cells) of tumor cells treated with either DMSO (vehicle control), or different concentrations of docetaxel or paclitaxel for 24h. *** indicates $p<0.001$, ns indicates $p>0.05$.

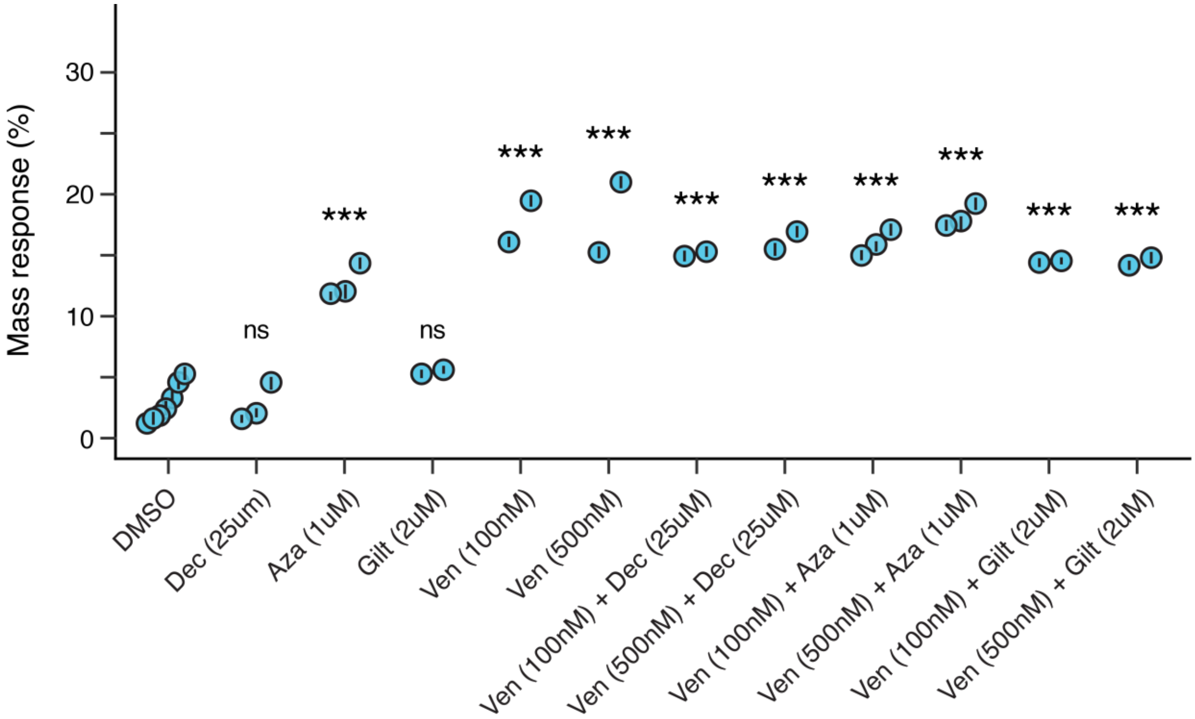

Supplementary Figure 11 | Primary acute myelogenous leukemia drug response measurements

Drug response data for an acute myelogenous leukemia blood specimen, which had a yield of ~19 million CD33+ cells following isolation. Data shown demonstrates mass response of 26 replicate conditions across 11 different drug conditions. Tumor cell measurements showed significant mass response to treatment with all HMA + venetoclax combinations, including decitabine + venetoclax, and to venetoclax alone. After the patient initiated therapy, they saw a marked reduction in bone marrow aspirate blast percentage following treatment with decitabine + venetoclax. *** indicates p<0.001, * indicates p<0.05. Points in mass response plot indicate independent instruments, error bars indicate 95% confidence interval for mass response.

Supplementary Note 1 | Primary specimen composition considerations

Primary specimens present distinct data collection challenges that must be addressed to ensure accurate interpretation of mass response following drug treatment. Specifically, when compared with cell lines, single-cell populations derived from primary specimens are more likely to contain a heterogeneous composition of cell types including immune, endothelial, and stromal cells in addition to the tumor cells of interest. These background cell populations have the effect of changing the underlying fraction of tumor cells and, as demonstrated in **Figure 6b** and **Supplementary Figure 3c**, could significantly alter the resulting mass response readout. Although image curation can improve the ability to identify cells of interest versus background debris and cellular aggregates, this approach alone does not currently have sufficient accuracy to distinguish between tumor and non-tumor cells within a population being measured. It is therefore crucial to account for this specimen heterogeneity upstream of mass response measurements. As described in the **Methods**, cell enrichment approaches based on magnetically activated cell sorting to either remove background cells (negative selection) or directly isolate tumor cells of interest (positive selection) can significantly reduce the effects of contaminating non-tumor cell populations. As this assay is implemented clinically, it will be important to verify the efficacy of these enrichment approaches as part of the routine quality control assessment of each specimen. For instance, flow cytometric assessment of enriched tumor cell populations to determine the relative fraction of tumor and non-tumor cell populations (e.g. EpCAM+/CD45- versus EpCAM-/CD45+ cells constituting tumor and immune cells, respectively, in an enriched malignant fluid specimen) can be used to determine if a cell population is of sufficient purity to assess tumor cell drug response. Additionally, as demonstrated in **Figure 3b**, various cell types often have distinctly different mass distributions. Future work will focus on incorporating these unique cell-type dependent single-cell mass distributions to further refine mass measurement data sets to focus only on tumor cells of interest.

Another key challenge of working with primary specimens is a loss of cellular viability *ex vivo*. The presence of dead cells within a population being tested artifactually increases the fraction of non-responding cells. As described in **Supplementary Figure 3c**, this background of non-responsive cells limits the ability to register a statistically significant mass response and therefore may lead to false negative results for low-viability tumor cell samples. To mitigate this risk, we have developed sample format-specific shipping protocols that rely on temperature control in transit and the use of preservative solution, where appropriate, to improve cell viability maintenance upstream of sample processing (**Methods**). These approaches demonstrated encouraging success for the specimens presented in **Figure 7**, with tumor cell viabilities of 88% for the blood sample, 96% for the bone marrow sample, 96% for the lung mass FNA, 99% for the soft tissue mass FNA, 95% for the neck mass FNA, and 75% for the pleural effusion sample, as determined by Trypan blue exclusion measurements on the purified tumor cell populations. For specimens with lower initial viabilities, commercially available dead cell removal kits (e.g. by means of magnetically activated cell sorting) can also be used to limit the influence of a dead cell background. Together, these sample shipment and viable cell enrichment strategies can be used to minimize the effects of cell viability loss on mass response signal interpretation. Nonetheless, in a clinical testing environment, it will still be prudent to collect cell viability measurements prior to cell drug response testing to guide sample acceptance and quality assurance.
