# Supplementary material for: A pipeline for malignancy and therapy agnostic assessment of cancer drug response using cell mass measurements
Source: Commun Biol. 2022 Nov 26;5:1295. doi: 10.1038/s42003-022-04270-3 (PMC9701192; doi:10.1038/s42003-022-04270-3)
Supplement: Supplementary file 5 — Reporting Summary [file 42003_2022_4270_MOESM5_ESM.pdf]

nature portfolio

Corresponding author(s): Robert Kimmerling, Clifford Reid

Last updated by author(s): November 9, 2022

Reporting Summary

Nature Portfolio wishes to improve the reproducibility of the work that we publish. This form provides structure for consistency and transparency in reporting. For further information on Nature Portfolio policies, see our Editorial Policies and the Editorial Policy Checklist.

Statistics

For all statistical analyses, confirm that the following items are present in the figure legend, table legend, main text, or Methods section.

n/a	Confirmed	
☐	☒	The exact sample size (*n*) for each experimental group/condition, given as a discrete number and unit of measurement
☐	☒	A statement on whether measurements were taken from distinct samples or whether the same sample was measured repeatedly
☐	☒	The statistical test(s) used AND whether they are one- or two-sided<br>*Only common tests should be described solely by name; describe more complex techniques in the Methods section.*
☒	☐	A description of all covariates tested
☐	☒	A description of any assumptions or corrections, such as tests of normality and adjustment for multiple comparisons
☐	☒	A full description of the statistical parameters including central tendency (e.g. means) or other basic estimates (e.g. regression coefficient) AND variation (e.g. standard deviation) or associated estimates of uncertainty (e.g. confidence intervals)
☐	☒	For null hypothesis testing, the test statistic (e.g. *F*, *t*, *r*) with confidence intervals, effect sizes, degrees of freedom and *P* value noted<br>*Give P values as exact values whenever suitable.*
☒	☐	For Bayesian analysis, information on the choice of priors and Markov chain Monte Carlo settings
☒	☐	For hierarchical and complex designs, identification of the appropriate level for tests and full reporting of outcomes
☒	☐	Estimates of effect sizes (e.g. Cohen's *d*, Pearson's *r*), indicating how they were calculated

Our web collection on statistics for biologists contains articles on many of the points above.

Software and code

Policy information about availability of computer code

Data collection	Flow Cytometry: Miltenyi MACSQuantify Software; Single-cell mass measurements: Custom software built in LabVIEW (18.0)
Data analysis	Flow Cytometry: FlowJo v10.8.1; Single-cell mass measurements: Custom analysis code written in R (4.1.2); Image classification: Custom analysis code written in Python (3.7.7) using the Keras library (2.3.1)

For manuscripts utilizing custom algorithms or software that are central to the research but not yet described in published literature, software must be made available to editors and reviewers. We strongly encourage code deposition in a community repository (e.g. GitHub). See the Nature Portfolio guidelines for submitting code & software for further information.

Data

Policy information about availability of data

All manuscripts must include a data availability statement. This statement should provide the following information, where applicable:

- Accession codes, unique identifiers, or web links for publicly available datasets
- A description of any restrictions on data availability
- For clinical datasets or third party data, please ensure that the statement adheres to our policy

The datasets generated during and/or analyzed during the current study are available from the corresponding author on reasonable request.

Human research participants

Policy information about studies involving human research participants and Sex and Gender in Research.

Reporting on sex and gender	*Use the terms sex (biological attribute) and gender (shaped by social and cultural circumstances) carefully in order to avoid confusing both terms. Indicate if findings apply to only one sex or gender; describe whether sex and gender were considered in study design whether sex and/or gender was determined based on self-reporting or assigned and methods used. Provide in the source data disaggregated sex and gender data where this information has been collected, and consent has been obtained for sharing of individual-level data; provide overall numbers in this Reporting Summary. Please state if this information has not been collected. Report sex- and gender-based analyses where performed, justify reasons for lack of sex- and gender-based analysis.*
Population characteristics	*Describe the covariate-relevant population characteristics of the human research participants (e.g. age, genotypic information, past and current diagnosis and treatment categories). If you filled out the behavioural & social sciences study design questions and have nothing to add here, write "See above."*
Recruitment	*Describe how participants were recruited. Outline any potential self-selection bias or other biases that may be present and how these are likely to impact results.*
Ethics oversight	*Identify the organization(s) that approved the study protocol.*

Note that full information on the approval of the study protocol must also be provided in the manuscript.

Field-specific reporting

Please select the one below that is the best fit for your research. If you are not sure, read the appropriate sections before making your selection.

☒ Life sciences ☐ Behavioural & social sciences ☐ Ecological, evolutionary & environmental sciences

For a reference copy of the document with all sections, see nature.com/documents/nr-reporting-summary-flat.pdf

Life sciences study design

All studies must disclose on these points even when the disclosure is negative.

Sample size	The minimum number of single-cell measurements required to determine drug response was determined using simulations of various numbers of cells drawn randomly from a population (as described in Supplementary Figure 3).
Data exclusions	Mass measurements were excluded from drug response assessment if classified as either 'debris' or 'aggregate' with the CNN image classification (as described in Figure 3).
Replication	When feasible, all single-cell mass measurements were conducted on multiple independent SMR instruments to verify reproducibility. Instrument to instrument concordance was also tested explicitly for multiple drug response measurements, as described in Supplementary Figure 1.
Randomization	There were no samples/participants randomized in this work.
Blinding	There was no group allocation that required blinding in this work.

Reporting for specific materials, systems and methods

We require information from authors about some types of materials, experimental systems and methods used in many studies. Here, indicate whether each material, system or method listed is relevant to your study. If you are not sure if a list item applies to your research, read the appropriate section before selecting a response.

Materials & experimental systems

n/a	Involved in the study
☒	☐ Antibodies
☐	☒ Eukaryotic cell lines
☒	☐ Palaeontology and archaeology
☒	☐ Animals and other organisms
☒	☐ Clinical data
☒	☐ Dual use research of concern

Methods

n/a	Involved in the study
☒	☐ ChIP-seq
☐	☒ Flow cytometry
☒	☐ MRI-based neuroimaging

Eukaryotic cell lines

Policy information about cell lines and Sex and Gender in Research

Cell line source(s)	ATCC: A549 (Male), MDA-MB-361 (Female), HT-29 (Female), NCI-H1666 (Female), NCI-H2228 (Female), MM.1S (Female), MM.1R (Female), NCI-H929 (Female), U266 (Male). ECACC: PC-9 (Male). DSMZ: PA-TU-8902 (Female)
Authentication	STR analysis performed by manufacturers.
Mycoplasma contamination	All cell lines tested negative for mycoplasma on a monthly basis.
Commonly misidentified lines (See ICLAC register)	*Name any commonly misidentified cell lines used in the study and provide a rationale for their use.*

Flow Cytometry

Plots

Confirm that:

☒ The axis labels state the marker and fluorochrome used (e.g. CD4-FITC).

☒ The axis scales are clearly visible. Include numbers along axes only for bottom left plot of group (a 'group' is an analysis of identical markers).

☒ All plots are contour plots with outliers or pseudocolor plots.

☒ A numerical value for number of cells or percentage (with statistics) is provided.

Methodology

Sample preparation	Flow cytometry was used to assess cell viability for various cell lines and single-cell suspensions isolated from primary tumor specimens. In all cases, cells were stained with FITC Annexin V (BioLegend, CAT #: 640945) and DAPI (BioLegend, CAT #:422801) following the manufacturers protocols.
Instrument	Miltenyi MACSQuant 10
Software	Collection: Miltenyi MACSQuantify, Analysis: FlowJo v10.8.1
Cell population abundance	No cell sorting was conducted
Gating strategy	Cells were gated using preliminary SSC/FSC assessment followed by single-cell gating using FSC-A/FSC-H linearity gating. Finally, viability assessment was performed with four quadrant gating of AnnexinV/DAPI. Cell cycle gating was performed with DAPI and BrdU, identifying populations of G1/G0, S, or G2/M cells.

☒ Tick this box to confirm that a figure exemplifying the gating strategy is provided in the Supplementary Information.
